# Diffraction-based approach for real-time monitoring of nanosecond direct laser interference patterning structure formation on stainless steel

**DOI:** 10.1038/s41598-024-60420-z

**Published:** 2024-04-26

**Authors:** Ignacio Tabares, Marcos Soldera, Bogdan Voisiat, Andrés Fabián Lasagni

**Affiliations:** 1 Institut für Fertigungstechnik, Technische Universität Dresden, George-Bähr Str. 3c, 01069 Dresden, Germany; 2 Fraunhofer Institut für Werkstoff und Strahltechnik IWS, Winterbergstr. 28, 01277 Dresden, Germany

**Keywords:** Engineering, Materials science, Optics and photonics

## Abstract

Direct Laser Interference Patterning (DLIP) stands out as a versatile and cost-effective method for functionalizing material surfaces at high throughputs. Monitoring the dynamics of the structure formation can lead to a deeper understanding of the interplay between the main factors governing the process and ultimately to optimize the final texture. Here, the formation of gratings on stainless steel by DLIP with ns-pulses is studied using a diffraction-based approach, which measures the time-resolved reflectivity (TRR) of the sample. Measurements are performed for single pulses across different laser fluences. The melting dynamics are analyzed and compared with numerical results. By correlating the recorded signals with the structure depths, growth rates of 11 nm/ns and 57 nm/ns were estimated for fluences of 1.9 J/cm^2^ and 5.3 J/cm^2^, respectively. Furthermore, two growth regimes are identified. In the fast growth phase, the melting time increased from 73 to 380 ns for fluences of 1.9 J/cm^2^ and 5.9 J/cm^2^, respectively, showing a good agreement with the performed thermal simulations.

## Introduction

The properties of a surface are influenced not only by its chemical composition but also by its topographical structure. Nature presents several examples where both characteristics are controlled, for example, the self-cleaning properties of the lotus leaf surface or the superhydrophobicity of some insect wings^1–3^. Also the improved hydrodynamic and tribological behavior found in the skin of sharks^4^ and snakes, respectively, that facilitate their motion^5^. Furthermore, in optics, the topography of surfaces the reflection, transmission and scattering of light, which can be implemented for example in optical devices^6^. Controlling the surface topography of devices can be also applied in biology. In this frame, producing repetitive (periodic) surface structures leads to controlled cell adhesion, proliferation, and differentiation^7,8^.

To produce technical surfaces exploiting the benefits provided by the above mentioned nature-inspired examples, different methods can be employed, including micro-milling processes^9^, electron and ion beam lithography^10–12^ and also laser-based techniques^13–15^. In the last case, Direct Laser Interference Patterning (DLIP) has shown to be a cost-effective technology due to its ability to produce nano/micro features with different geometries (for example lines or dots) and on a wide range of materials^16,17^. This technique can also be applied to complex 3D parts and not only planar substrates like most conventional methods^18^. The DLIP method is based on the overlap of two or more laser beams onto the surface producing an interference pattern, which can ablate or melt the material at the maxima positions, thus forming a periodic grating on the material^19^.

When coupled to a nanosecond laser source (ns-laser), typically the material is locally molten and flows from the maxima to the minima positions with subsequent re-solidification^20^. One of the processes governing the dynamics of melt flow is known as Marangoni convection^21,22^, which takes places when a non-uniform temperature distribution exists in a fluid. Due to the laser intensity contrast in the interference pattern, significant thermal gradients (e.g. over 1000 K/µm) are present between maxima and minima positions, leading to gradients in the surface tension across the molten pool^23^. In addition, when applying nanosecond pulses, also another phenomenon called recoil pressure plays a key role in structure formation dynamics. In particular, in the case that material evaporation takes place, the vapor (and plasma) produce a recoil force over the interference maxima positions, pushing the molten material further away to the interference minima^21,24^. In summary, at lower fluences the Marangoni convection emerges as the primary mechanism governing the redistribution of molten material during nanosecond laser pulses, while at higher intensities, the dominant influence shifts to the recoil pressure effect^21,25^.

Although several research works have been published related to the processing of different metallic surfaces using ns-DLIP (e.g. steels^26^, titanium^27^ and aluminum alloys^28^, nickel^29^), no research has been conducted according to the knowledge of the authors for retrieving information about the process of structure formation experimentally. This can enable a better understanding of the dynamics involved in the process as well as permitting a better control of the topographies that can be produced. The performed experimental work has been so far dedicated to semiconductors (Si and Ge) which were processed with a two-beam DLIP setup with 20 ns pulses^30,31^ and 8 ns^32^. In addition, different simulation models and ex-situ measurement have been performed for metals, in order to estimate for instance the amount of molten and vaporized material as well as estimating temperature distributions^33^. Furthermore, in a recent publication by T. Jähnig et al. it was investigated the influence of the sulphur (S) content in different steels (from 30 to 3000 ppm) in the melt-dynamics during ns-DLIP, in particular considering the effects of the mentioned component in the surface tension gradients (e.g. positive or negative Marangoni’s convection)^34^. The performed simulation permitted the determination of the flow dynamics of the material within the molten pool, but could not retrieve information about the process dynamics of structure formation since the surface of the simulated area was fixed. In addition, the effect of recoil pressure was not considered, since only low laser fluences were employed.

In case of laser-based surface treatments, there are different methods that have been developed to evaluate laser-matter interaction processes (laser-induced damage, plasma formation, phase transitions, laser-induced chemical reactions) which occur within a laser pulse^35^. These methods include Spot Probing^36^, Diffraction Probing^37^, Coherent Scattering^38^, fs-microscopy^39^ among others. In particular, in spot probing, the variation of the optical reflectivity can be used to assess phase changes on the surface of a material. This method has played a central role in performing time sensitive measurements during pulsed-laser annealing of semiconductors, e.g., determining melting thresholds in silicon and germanium^39,40^. The method has also been applied to metallic substrates for example for determining melting duration and melting thresholds in copper, gold and nickel films^41^ as well as bulk metals such as stainless steel or titanium with accurate results when compared with IR radiometry measurements^42^. Recently, this optical probing method has been implemented for monitoring the formation of gratings in semiconductors using single pulse nanosecond two-beam DLIP with favorable results for determining directly their topography as well as providing an insight on structure heights for structure depths below 1 µm^43^.

In this work, a reflectivity-based method, which relies on a simplified concept of the optical probing method, is used for the first time in stainless steel to determine experimentally different features in ns-DLIP single pulse processing, including melt formation as well as structure formation dynamics. A correlation between the reflectivity signal and structure depth is obtained allowing the estimation of the structure depth evolution during the first hundreds of nanoseconds after the laser was fired. After the structuring process, the line-like fabricated structures are characterized using confocal microscopy. To assess the validity of the obtained experimental results, a thermal simulation model based on heat diffusion is used for retrieving information on the dependence of the melting time with the applied laser fluence. Finally, a simple calculation is presented, permitting the calculation of the growth rate of the periodic structures depending on the laser fluence.

## Materials and methods

The material of choice for this study was AISI 304 stainless steel. This alloy is proven to be of relevance for several industrial sectors such as pharmaceutical, energy and food industry^44–47^. The samples consist on plates with a thickness of 1 mm and dimensions of 80 mm × 60 mm. The substrates were electropolished to a roughness Sa of 15 ± 1 nm and cleaned with ethanol previous to the laser-treatment. Initial roughness of the sample was assessed using confocal microscopy by analyzing an area of 1.67 mm by 1.25 mm. The measurement was performed 5 times in different spots of the plate to obtain a representative value of the material roughness.

**Figure 1:**
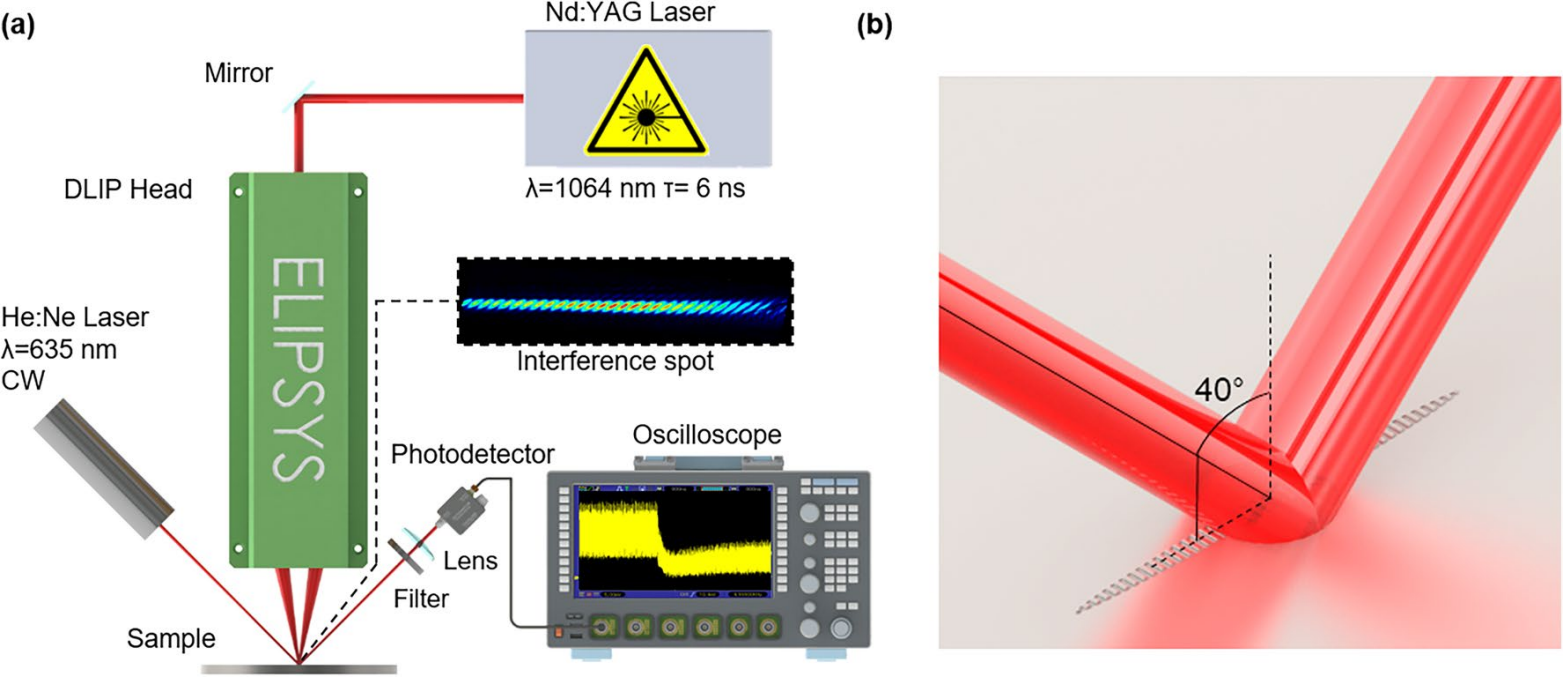
(**a**) Experimental ns-DLIP structuring set-up and time-resolved reflectivity configuration. (**b**) Representation of the measuring setup beam over the DLIP spot with its characteristic angles depicted.

The fabrication of the periodic structures by Direct Laser Interference Patterning (DLIP) was performed using a Q- switched diode-pumped slab-type solid-state Nd:YAG laser (InnoSlab IS400-3-GH, Edgewave GmbH, Germany) with a pulse duration of 6 ns and a fundamental wavelength λ of 1064 nm. The laser beam was guided with mirrors to a DLIP head (ELIPSYS^®^, SurFunction GmbH, Germany). This optical configuration is capable of splitting the beam by means of a diffractive optical element (DOE) and shaping two of the resulting sub-beams by using an array of lenses in order to produce an interference spot characterized by parallel fringes on the sample. The spot has an elongated geometry with an approximately elliptical shape with major (a) and minor (b) axis radii of 1300 µm and 65 µm, respectively. For calculating the average laser fluence F, first the average power P of the DLIP modulated beams on the sample position was measured with a powermeter (FieldMaxII-TO and sensor PM1K+ , Coherent, United States) at a laser repetition rate *f* = 5 kHz and different laser output powers. Then, the pulse energy *E*_p_ = *P*/*f* was calculated. Next, the fluence *F* was determined considering the elliptical shape of the elongated laser spot as *F* = *E*_p_/(π × a × b). The time-resolved reflectivity experiments were performed by setting the structuring laser at a nominal repetition rate of 5 kHz to obtain the same pulse energies as in the laser power measurements. The line-like pattern is characterized by a spatial period (distance between interference maxima positions) of 6.0 µm. The experimental setup is depicted in Fig. 1a. Processing of the samples was performed by firing single laser pulses onto the metallic sample with varying laser fluence from 1.9 to 5.9 J/cm^2^. For each fluence, five individual pulses were recorded for statistical significance.

An optical measuring setup was assembled for the time-resolved reflectivity (TRR) measurements. The TRR measuring system utilized as probing light a continuous wave He:Ne laser (Model 1135, Uniphase, United States) with a central wavelength of 635 nm, a circular spot with a diameter of 690 µm and 9 mW of power. The beam was directed into the central part of the processing zone demarked by the interference spot dimensions, with an azimuthal angle perpendicular to the pattern fringe direction and at an elevation angle of 40° (see Fig. 1b). This geometry (which is not following the optical axis of DLIP head and thus of the plasma plume) permits the reduction of possible absorption effects of the He:Ne laser by the plasma. The specular reflected light propagated through a narrow band interference filter (FLH635-10, Thorlabs GmbH, Germany) to suppress any contribution of reflected light emitted by the Nd:YAG structuring laser and also to limit the effects of the plasma plume interaction with the measurement. After the filter, the beam was focused with a f = 100 mm plano-convex lens into a Si photodetector (DET025AFC/M, Thorlabs GmbH, Germany). The photodetector signal was acquired by an oscilloscope (Lecroy WavePro W7100A) operating at 1 GHz (with 10 GS/s, 400 ps rise time). The oscilloscope was synchronized with the laser controller’s triggering signal in order to acquire the complete temporal evolution of the reflectivity for a single pulse. No normalization of the values has been performed and as far as this work is concerned, the output signal of the photodiode—which captures the intensity of the specular reflection of the zeroth order of diffraction—was considered.

The topography of the laser-treated surfaces was measured by confocal microscopy (Sensofar S Neox 3D Surface profiler, Sensofar Metrology, Spain) equipped with a 150 × objective with a lateral and vertical resolution of 0.14 µm and 1 nm, respectively. The measurements were taken in the center of the track and the resulting images were analyzed utilizing SensoMap 7 software (Sensofar, Barcelona, Spain). From each track, two profiles were extracted to perform the structure depth measurements. The surface roughness values for the DLIP structures were calulated with the SensoMap software.

In addition to the laser experiments, a 2D thermal model based on Finite Element Method (FEM) was implemented in order to estimate the time over which the material is molten. The simulations were performed using the FlexPDE^®^ software (PDE Solutions, USA) and is based on the heat diffusion equation:

1 $$\rho {c}_{p}\frac{\partial T(x,z,t)}{\partial t}={q}_{a}-{q}_{m}-{q}_{v}+\nabla \left(k\nabla T\left(x,z,t\right)\right),$$

where *T* is the temperature at the position (*x*: parallel to the material surface, *z*: perpendicular to the surface) and time *t*, *q*_*a*_ the added heat due to the laser radiation and *q*_*v*_ the heat required to vaporize the metal. The symbols *c*_*p*_, *k*, and *ρ* stand for the specific heat, the thermal conductivity and the density, respectively. For the simulations, the following assumptions were made:

I. No radiation loss from the surface,
II. No convection above the surface,
III. No convection due to gravitational or electromagnetic effects upon melting of the material,
IV. The laser energy distribution is considered as 5 periods of a sinusoidal function including a Gaussian distribution in time,
V. Solid–liquid and liquid–vapor transitions are considered in the heat conduction problem, as well as latent heats of fusion and vaporization.

Further information about the heat transfer model applied in this paper has already been published elsewhere^21,25^.

## Results and discussion

### DLIP single pulse structuring and topographical characterization

**Figure 2:**
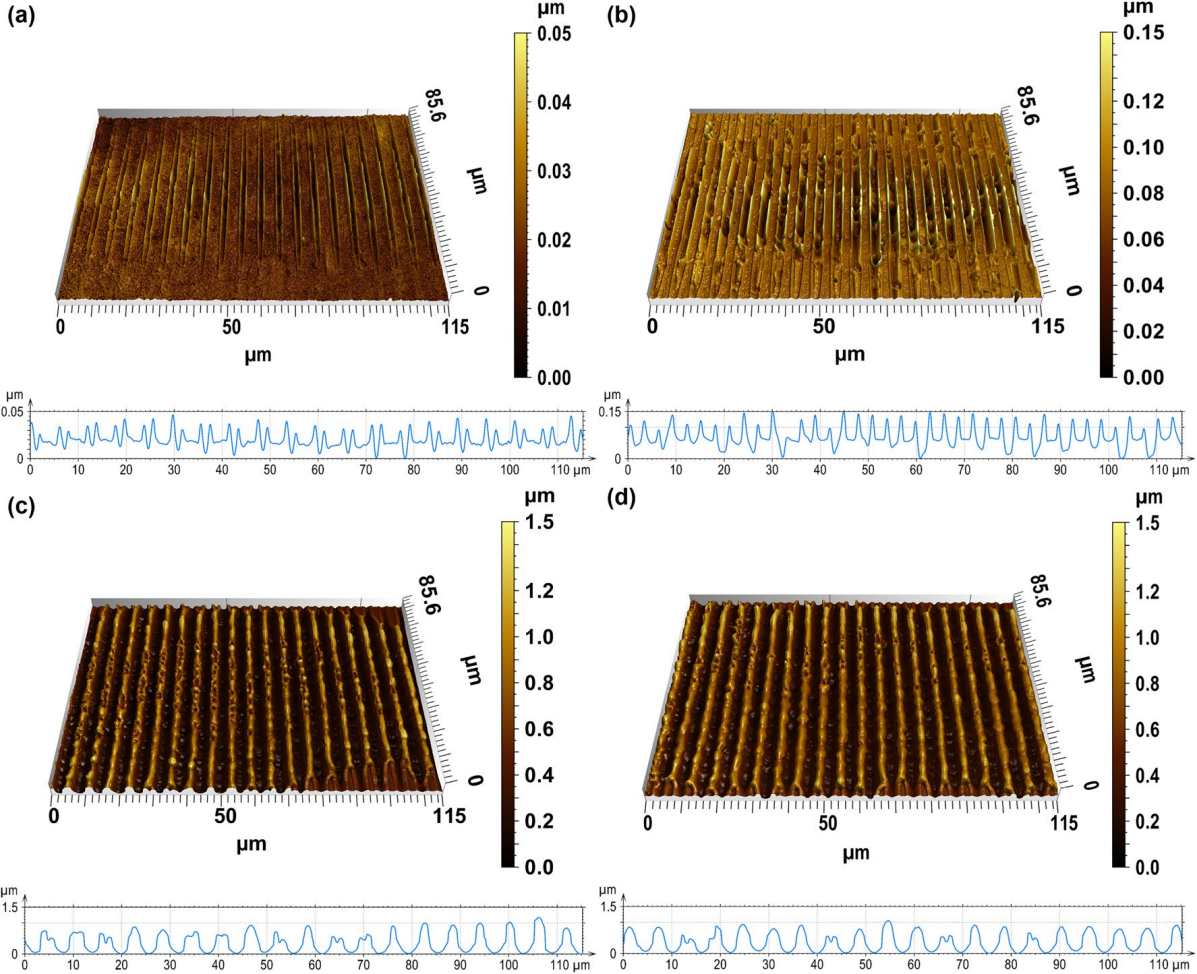
Confocal images of DLIP structures at the central region of the elongated spot. The used laser fluences were: (**a**) 1.9 J/cm^2^, (**b**) 2.3 J/cm^2^ (**c**) 4.2 J/cm^2^ (**d**) 5.3 J/cm^2^. Only one laser pulsed was used.

The processing of the metallic samples by DLIP using an elongated spot led to the fabrication of line-like periodical features with a spatial period of 6.0 µm. Exemplary confocal microscope images of the produced patterns at different laser fluences can be observed in Figure 2 (note that the scale bars for the structure depth are intentionally different, for a better visualization of the results). For the lowest fluence of 1.9 J/cm^2^ (Figure 2a), the grooves are barely detectable, having an average structure depth of only 15 nm. In case of the laser spot processed at 2.3 J/cm^2^ (Figure 2b), the structure depth was also relative low, with an average value of 27 nm.

As it can be seen for these fluences (1.9 J/cm^2^ and 2.3 J/cm^2^), the energy density was high enough to induce the melting of the substrate at the interference maxima positions, but it was not high enough to completely drive the melt towards the minima positions and thus resulting in a double peak structure, which has been already reported in previous research works^25^. Differently, for fluences of 4.2 J/cm^2^ and 5.3 J/cm^2^ (Figure 2c and d, respectively) the molten material at the maxima positions was totally displaced towards the minima and solidified, reaching higher structure depths (e.g. 733 nm for a laser fluence of 5.3 J/cm^2^).

A further increase of the laser fluence (up to 5.9 J/cm^2^) produced more molten material, being also able to flow outward the maxima positions leading to deeper structures. At this laser fluence values, this effect is known to be driven by a combination of Marangoni convection as well as the recoil pressure as previously described^21^.

**Figure 3:**
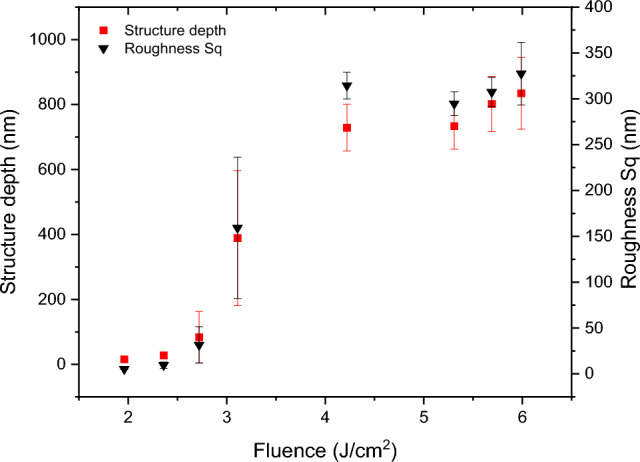
Variation of structure depth and mean square surface roughness (S_q_) with applied laser fluence.

The dependence of the structure depth as well as the mean square surface roughness (S_q_) was plotted as function of the applied laser fluence as depicted in Figure 3. The reported values correspond to the measurements performed after a single laser pulse was delivered to the sample. From the plot, two well-defined regimes can be identified. It can be observed that both the structure depth and surface roughness continuously increase as the fluence increase from 1.9 to 2.7 J/cm^2^ (implying an increase in the structure depth from 15 to 83 nm). This first regime is followed by a transition zone were small increments in laser fluence lead to a signifcant increase in the surface roughness, as previously reported elsewhere^21,25^. For laser fluences over 4 J/cm^2^ a second regime can be identified, in which the maximal value for both roughness S_q_ and structure depth was reached (e.g., for Sq ~ 327 nm) and does not increase significantly with an increase in the laser fluence. This can be related to an excess of melting at the maxima positions due to the high laser power applied, which does not further contribute to an increase of the structure depth^25^. The presented values in Figure 3 serve for developing a simple method of estimating the temporal evolution of the structure depth as it will be described in the next sections.

### Temporal evolution during single pulse DLIP experiments

In order to monitor the formation of the structures in DLIP during a single pulse, time-resolved reflectivity measurements were performed. As described in the experimental section, the specular reflectivity of the area irradiated by the probe laser (CW, He:Ne at 635 nm wavelength), which is undergoing the structuring process with the two-beam DLIP setup, is directed and captured by the photodetector. Because during the DLIP process a periodic grating is formed, the cw laser beam is diffracted into different diffractions orders^48^. In this work, the photodetector was located at the position of the zero diffraction order, which means that the changes observed in the captured signal can be produced by local changes of the reflectivity due to its dependency with temperature, and/or due to the formation of the periodic structure. The latter results in an increase of the intensity of the first diffraction orders (e.g., ± 1, ± 2) and thus in a reduction of the intensity corresponding to the zero diffraction order.

**Figure 4:**
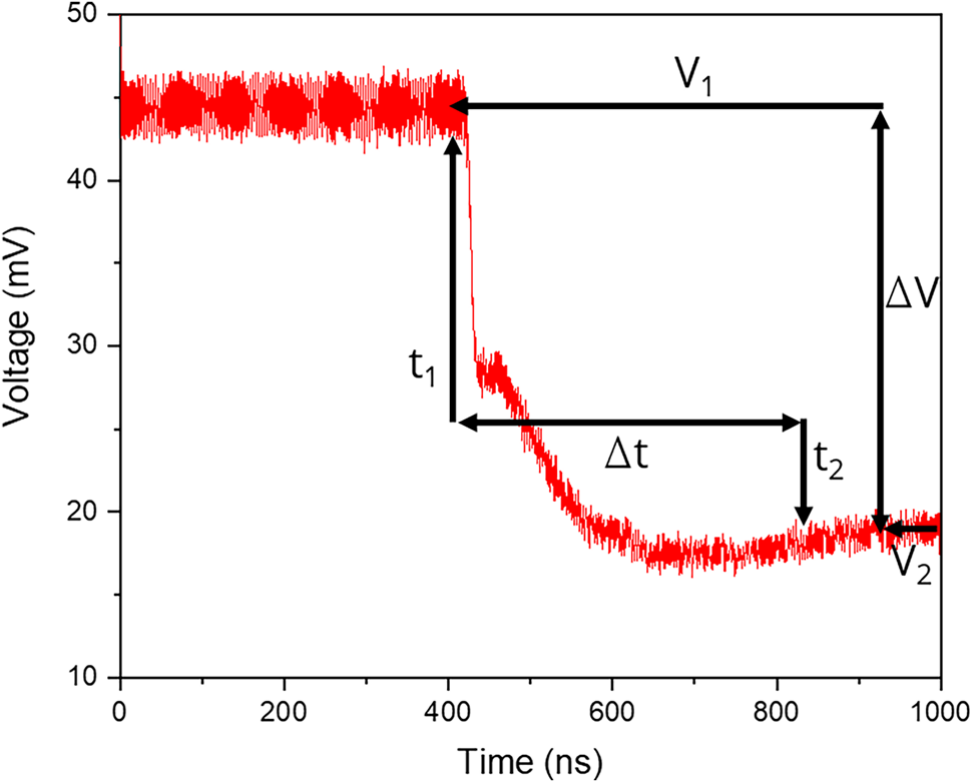
Time resolved reflectivity curve after FFT filtering. The curve corresponds to a stainless steel substrate irradiated at a laser fluence of 5.9 J/cm^2^. From the plot, both the melting time (Δt) as well as the relative change of the intensity of the reflected zero order (ΔV) can be read.

Figure 4 shows a typical TRR curve depicting the fundamental parameters for this analysis. A 100 points FFT filter was applied to the raw signal to reduce the high frequency noise. The voltage values V_1_ and V_2_ represent the initial and final reflectivity states, respectively, which permitted determining the intensity variation of the zero order. This variation is proportional to the measured voltage difference ΔV. The laser firing time is not indicated in the curves but lies in the vicinity (within a few ns) to the position where the voltage signal drops steeply^41,43^. On the temporal axis, the coordinates t_1_ and t_2_ were determined for each experiment, and they indicate the beginning and end of the molten state of the thermalization process, respectively. The time t_1_ can be identified in the curve as the time the reflectivity signal drops abruptly due to the material fusion. The time t_2_ can be linked to the end of the resolidification process as reported in the investigations by Martan et al. on stainless steel^42^. From their results, the time t_2_ can be identified in the curves as the inflection point after the signal minimum. Thus, the difference between both values (Δt) stands for the interval in which the material remains molten (melting time). The validation of these assumptions are discussed later in the course of this work when the thermal simulations are presented.

**Figure 5:**
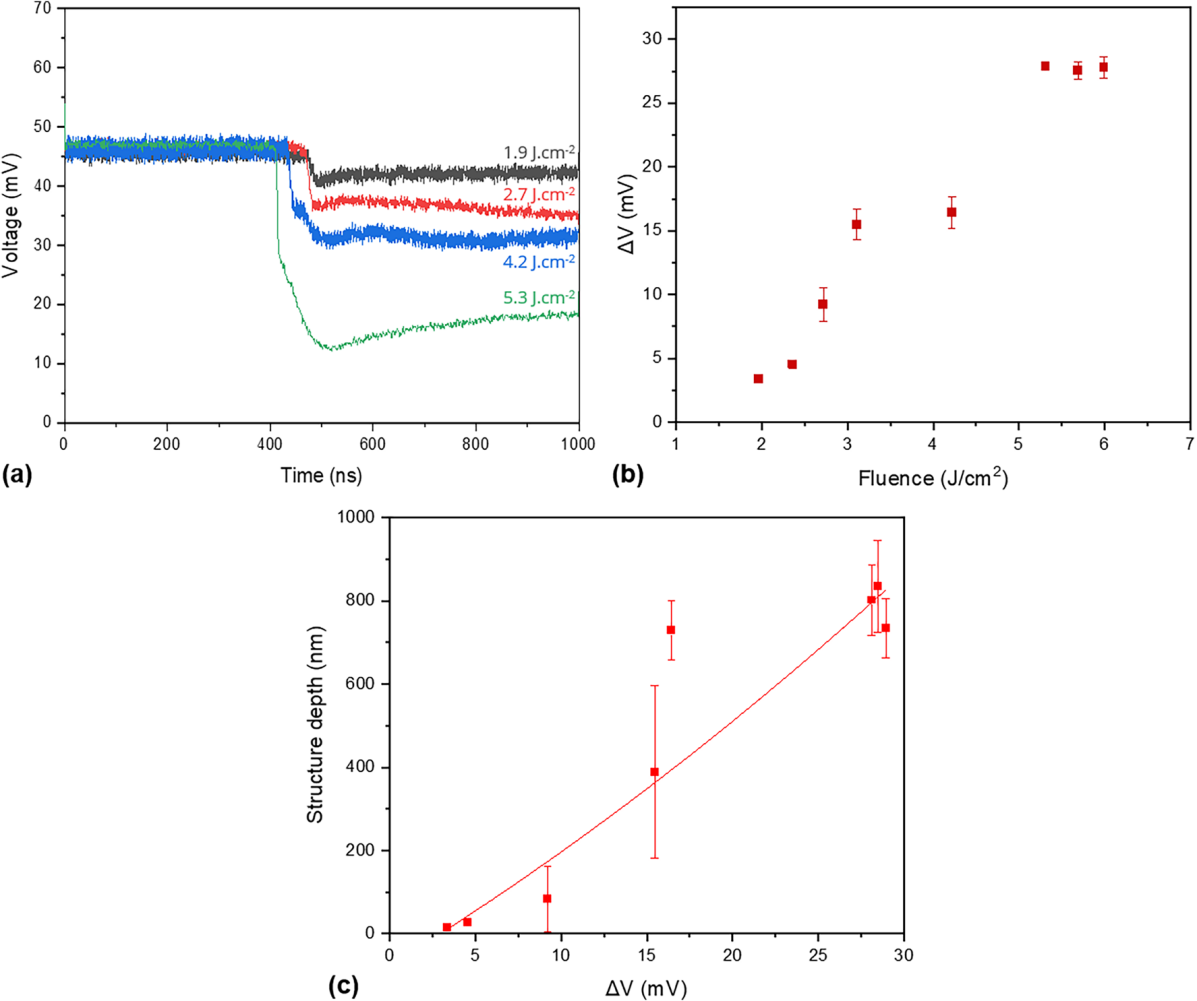
(**a**) Time-resolved reflectivity curves of stainless steel samples irradiation at different laser fluences. (**b**) Difference in starting and final reflectivity (voltage) after each pulse as function of laser fluence. (**c**) Variation of structure depth (from Fig. 1) dependency with the voltage drop at the end of the single pulse event.

The behavior described in Fig. 4 was also generally observed when using different laser fluences as it is shown in Fig. 5a. During the first ~ 400–450 ns, the measured voltage can be linked to the initial reflectivity value of the untreated stainless steel surface (~ 45–47 mV). After that, a fast and strong decrease in the voltage signal was recorded, in a time range from 10 to 15 ns (depending of the applied laser fluence), denoting strong structural changes. After that, the signal further decreased which means that the depth of the produced periodic structures further increased, and finally the reflectivity reached the steady state condition.

In case of the samples irradiated at high laser fluences (see for 5.3 J/cm^2^), the final intensity of the zero diffraction order was slightly higher than during the time in which the material was partially molten. This effect can be explained by two possible reasons: the increase in the voltage signal can be attributed to an increase of the reflectivity of the material when cooling (as reported in^42^ for stainless steel 17246), or due to a decrease in the structure depth. In the last case, a recent publication by Heinrich et al. which focused on numerical simulations of the structure formation in three-beam DLIP, reported on such possible temporal morphological changes^49^.

From the TRR curves, it was also possible to see that samples irradiated at higher laser fluences resulted in higher intensity changes in the zero diffraction order (ΔV), as shown in Fig. 5b. In addition, when comparing these results with the structure depth dependency with the laser fluence (see Fig. 3) a similar behavior was observed. This denotes a possible correlation between ΔV with the structure depth, which is shown in Fig. 5c. Furthermore, these changes are in agreement with the findings reported in^48^, where the average depth of dot-like geometries produced by ns-DLIP treatment could be estimated based on the variation of the relative intensities of the zero and first diffraction orders.

**Figure 6:**
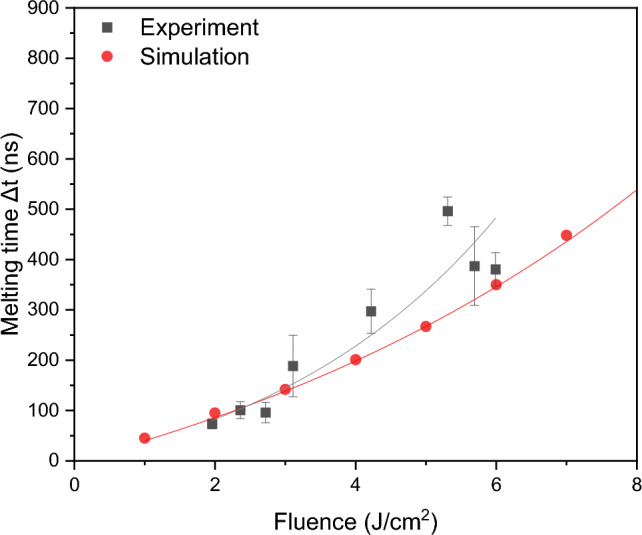
Simulated (red circles) and experimental (gray squares) melting times as function of the applied laser fluence (the line are just guides for the eye).

Regarding the melting time (Δt), this information was also calculated from the TRR curves, using the procedure previously described (see Fig. 4). The dependency of the melting time with the laser fluence is shown in Fig. 6. As it can be seen, higher fluence values led to prolonged melting times, attributed to the increased energy absorbed by the material.

### Validation of the probe investigations and structure depth evolution calculation

In order to validate the findings obtained with the time-resolved reflectivity (TRR) measurements, a thermal simulation was performed. From the numerical results, the characteristic times of the molten dynamics for representative fluence values covering the fluence range in this work were calculated. As mentioned in the materials and methods section, the simulation only permits to calculate temporal temperature distributions within the irradiated material as well as molten and vaporized regions. It must be also mentioned that the performed simulations are based on a simplification the interaction of an interference pattern with a metallic surface, and do not considering neither material flow nor transfer of mass from the melt pool. The objective is only to validate the order of magnitude of the calculated meting times compared to the experiments.

The simulated melting times (Δt) as function of the applied laser fluence are shown in Fig. 6. As it can be seen, the melting time increased from 95 to 350 ns for laser fluences of 2.0 J/cm^2^ and 6.0 J/cm^2^, respectively (see red circles). These values are in good agreement with the experimental measurements (see gray squares in Fig. 6). For instance, for 1.9 J/cm^2^ and 5.9 J/cm^2^ the experimental melting times were 73 ns and 380 ns, respectively. Furthermore, both the experimental and simulated values display similar tendencies. The differences observed can be attributed to the simplified assumptions that were considered^21,25^.

Finally, a polynomial fit, yielding an R^2^ value of 0.89 (see Fig. 5c), was applied to elucidate the correlation between the structure depth and the measured voltage difference obtained from the TRR experiments. This fitted model was then employed to predict the evolution of structure depth in single-pulse ns-DLIP experiments. For simplification of the model, the impact of temperature on reflectivity was excluded. Consequently, variations in the measured intensities for the zero diffraction order were solely attributed to changes in the structure depth induced by the diffraction grating being produced over the stainless steel metallic surface.

**Figure 7:**
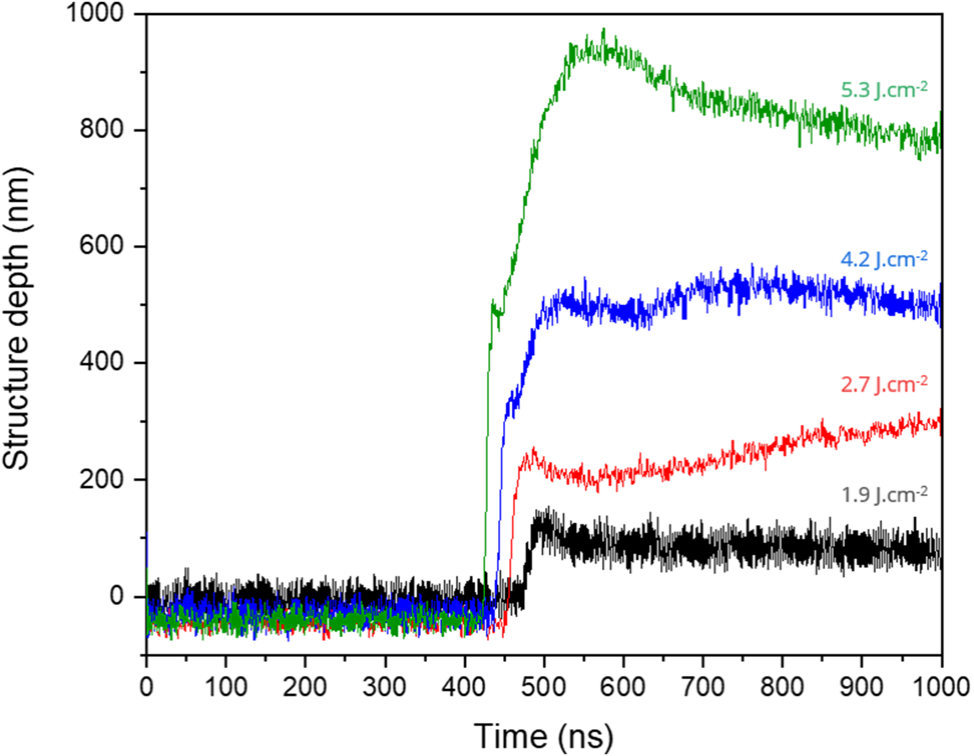
Structure depth evolution during ns-DLIP processing calculated from time-resolved reflectivity (TRR) (Fig. 5a) measurements and the polynomial fit describing the relationship between ΔV and the structure depth (Fig. 5c).

The used procedure consists first in subtracting the measured voltage before the laser pulse from the data already presented in Fig. 5a. Then, the polynomial fit can directly be implemented obtaining the time-dependent structure depth evolution. The results are presented in Fig. 7. The slope of the curve represents the velocity in which the structure grows and increases when higher laser fluences are applied. The curves exhibit two distinct regimes: an initial phase (regime 1) characterized by a sudden growth in the structure depth, followed by a subsequent phase (regime 2) marked by a more gradual rate of change. The first regime (fast growth) takes place during the first ns of the laser treatment (~ 7 to 9 ns, see Table 1) showing also a direct dependency with the applied laser fluence. For example, for 1.9 J/cm^2^ and 5.3 J/cm^2^ the line-like structures are formed at a speed of 11 nm/ns and 57 nm/ns, respectively. After this fast phase, the structure growth rate decreases up to a factor of 19, leading to speeds of ~ 3 to 4 nm/ns for all used fluences.

**Table 1:** Calculated growth rates depending on laser fluence for regimes 1 and 2. The numbers were calculated from Fig. 7.

Laser fluence (J/cm^2^)	Regime 1 (fast growth)			Regime 2 (slow growth)		
	Time (ns)	Total depth variation (nm)	Growth rate (nm/ns)	Time (ns)	Total depth variation (nm)	Growth rate (nm/ns)
1.9	10	115	11	–	–	–
2.7	8	175	22	23	82	~ 4
4.2	9	273	30	79	257	~ 3
5.3	7	400	57	144	541	~ 4

The above mentioned values (for the fast growth rate) are consistent with the simulations performed by Heinrich et al. for three-beam DLIP experiments on stainless steel, using a 10 ns laser source. For instance, for a laser fluence of 4.8 J/cm^2^, a growth rate of 59 nm/ns could be calculated. Regarding the decrease in the structure depth, in particular for the laser fluences of 5.3 and 4.2 J/cm^2^, further investigations are necessary. This behavior was observed in the FEM simulations reported in ^49^. In the published report, the authors also considered a DLIP process with ns-pulses and fluence levels similar as the ones presented in this work. It was also observed that the molten material forms structures in the first hundreds of ns, reaching a maximum structure depth and then slowly decreasing the depth due to gravity, surface tension, and Marangoni convection effects start to dominate until the material re-solidifies. In the light of this findings, it is necessary to further investigate the contribution of reflectivity variations due to the increased temperature. On the other hand, this simple approach shows to be very promising for obtaining experimental information regarding the formation of periodic structures using DLIP with ns pulses.

## Conclusions

This work introduces the application of an optical probing method to monitor the process of Direct Laser Interference Patterning when applied to stainless steel surfaces using 6 ns laser pulses. Periodic line-like structures with a period of 6.0 µm were produced using laser fluences ranging from 1.9 to 5.9 J/cm^2^ and one single pulse. Time-resolved reflectivity measurements permitted the experimental determination of the melting times of the material, depending on the employed laser fluence. Additionally, the growth rates of the resulting line-like structures were estimated through these measurements using a simplified model.

The following findings can be summarized from this work:

I. The melting times in ns-DLIP increased with the applied laser fluence, from 73 to 380 ns for 1.9 J/cm^2^ and 5.9 J/cm^2^, respectively.
II. These values can be also estimated from simple numerical simulations, obtaining a good agreement with the experimental results.
III. The variations in the temporal evolution can be used for estimating the growth rate of the line-like structures taking into consideration the intensity decrease for the zero diffraction order in DLIP.
IV. Two different regimes were identified during the structure formation process in DLIP, consisting on an initial phase characterized by a sudden growth in the structure depth, followed by a subsequent phase marked by a more gradual rate of change.
V. In the first regime, growth rates between 11 and 57 nm/ns were calculated, for laser fluences of 1.9 J/cm^2^ and 5.3 J/cm^2^, respectively.
VI. Regarding the regime with a lower growth rate, speeds of ~ 4 nm/ns could be calculated for the laser fluences of 2.7 J/cm^2^, 4.2 J/cm^2^ and 5.3 J/cm^2^, for the laser fluence of 1.9 J/cm^2^ a second growth rate regime could not be identified.

In the future, further work is necessary to investigate the contribution of reflectivity variations due to the increased temperature as well as comparing the behavior of other metallic surfaces. Finally, a simulation model including flow of material has to be developed, in particular for evaluating possible temporal decreases of the structure depth during the DLIP structuring process.

## Data Availability

Any supporting data for this article is available from the corresponding author upon reasonable request.
